# Optimal Design of the Austenitic Stainless-Steel Composition Based on Machine Learning and Genetic Algorithm

**DOI:** 10.3390/ma16165633

**Published:** 2023-08-15

**Authors:** Chengcheng Liu, Xuandong Wang, Weidong Cai, Jiahui Yang, Hang Su

**Affiliations:** 1 Institute of Structural Steel, Central Iron and Steel Research Institute, Beijing 100081, China; ustbliuchengcheng@foxmail.com; 2 Material Digital R&D Center, China Iron and Steel Research Institute Group, Beijing 100081, China; tony775316@163.com (X.W.); yjh7646@126.com (J.Y.)

**Keywords:** machine learning, austenitic stainless steel, genetic algorithm, composition optimization

## Abstract

As the fourth paradigm of materials research and development, the materials genome paradigm can significantly improve the efficiency of research and development for austenitic stainless steel. In this study, by collecting experimental data of austenitic stainless steel, the chemical composition of austenitic stainless steel is optimized by machine learning and a genetic algorithm, so that the production cost is reduced, and the research and development of new steel grades is accelerated without reducing the mechanical properties. Specifically, four machine learning prediction models were established for different mechanical properties, with the gradient boosting regression (gbr) algorithm demonstrating superior prediction accuracy compared to other commonly used machine learning algorithms. Bayesian optimization was then employed to optimize the hyperparameters in the gbr algorithm, resulting in the identification of the optimal combination of hyperparameters. The mechanical properties prediction model established at this stage had good prediction accuracy on the test set (yield strength: R^2^ = 0.88, MAE = 4.89 MPa; ultimate tensile strength: R^2^ = 0.99, MAE = 2.65 MPa; elongation: R^2^ = 0.84, MAE = 1.42%; reduction in area: R^2^ = 0.88, MAE = 1.39%). Moreover, feature importance and Shapley Additive Explanation (SHAP) values were utilized to analyze the interpretability of the performance prediction models and to assess how the features influence the overall performance. Finally, the NSGA-III algorithm was used to simultaneously maximize the mechanical property prediction models within the search space, thereby obtaining the corresponding non-dominated solution set of chemical composition and achieving the optimization of austenitic stainless-steel compositions.

## 1. Introduction

**Figure 1:**
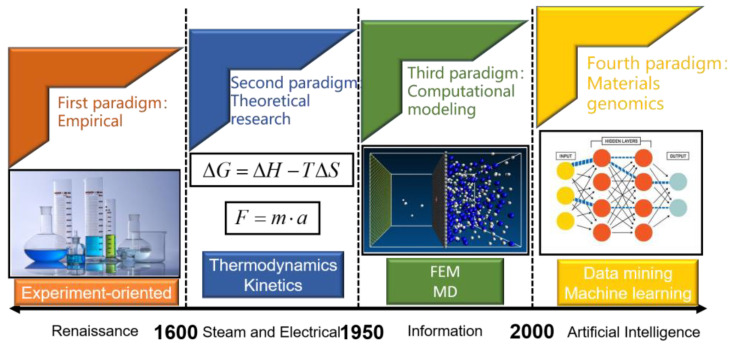
Evolution of material research and development methods.

Figure 1 shows the four stages of a material research and development model over recent history [1]. The first stage is the Empirical paradigm, which focuses on practical experiments in substances’ structure, properties, preparation processes, and applications by combining laboratory experience. This paradigm provides a large amount of quantitative data to support repeatable conclusions but lacks deep understanding of material properties in larger scale systems. The next stage is the Theoretical research paradigm, which mainly summarizes theoretical models from previous research based on experiments, forming various laws in mathematical equations. However, as systems become more complex, the calculations of theoretical models become inaccurate. With the advent of computers, the Computational modeling paradigm began using computer simulation techniques to study material properties, such as finite element models and molecular dynamics simulations. The advantage of this paradigm is that it can study problems that are difficult to achieve experimentally and quantitatively predict material properties, thus promoting the development of previous paradigms. However, the disadvantage is the strict requirements on the definition of simulation processes and selection of initial parameters. With further developments in computer science, the Materials genomics paradigm has emerged in the past 20 years. This paradigm integrates experimental design, computer simulation, and theoretical analysis methods to systematically obtain large-scale data of materials using high-throughput experiments, and after uses computer algorithms to analyze these data and deduce unknown material properties. The advantages of this paradigm are increased efficiency in material research and reduced trial-and-error efforts. Recently, this material research paradigm has been applied in various aspects, including material feature construction and selection [2,3,4,5], material organization and property prediction [6,7,8,9], discovery of explicit structure–effect relationships [10,11,12,13], and material optimization design [14,15,16], greatly promoting the development of material science.

Austenitic stainless steel is a type of steel material whose matrix is primarily composed of the face-centered cubic crystal structure of austenite. It has excellent corrosion resistance and mechanical properties and is widely used in high-service requirements such as deep-drawn forming components, as well as acid-corrosive medium pipelines, containers, and structural components. As the service environment of stainless steel continues to deteriorate, higher requirements are imposed on its performance, presenting significant challenges in designing new stainless steels that can meet these demands. Traditional “trial-and-error” methods suffer from high experimental costs, long experimental cycles, and low R&D efficiency. In contrast, machine learning can perfectly avoid these issues and achieve efficient material design. Research has shown that this method is reliable in designing new materials. Diao et al. [17] used machine learning to establish a mechanical performance prediction model for carbon steel. They used the strength-ductility product as the optimized target value and ultimately achieved the design of carbon steel with excellent comprehensive performance. Reddy et al. [18] combined neural networks and genetic algorithms to optimize the chemical composition and heat treatment parameters of medium carbon steel to achieve desired mechanical properties. Shen et al. [19] introduced physical metallurgy models into machine learning models, effectively improving the accuracy of machine learning predictions. Furthermore, the study showed that the introduction of physical metallurgy can improve the design accuracy and efficiency by eliminating intermediate parameters that do not follow physical metallurgical principles in the machine learning process.

In this study, experimental data on the compositional features, process features, testing features, and mechanical properties of austenitic stainless steel were collected as a dataset. By establishing a high-precision performance prediction model and using the range of compositional feature changes as the search space, genetic algorithms were used to search for optimization in this space. The objective was to optimize the composition of austenitic stainless steel and improve the R&D efficiency of high-performance steel materials.

## 2. Materials and Methods

### 2.1. Optimal Design Strategy

**Figure 2:**
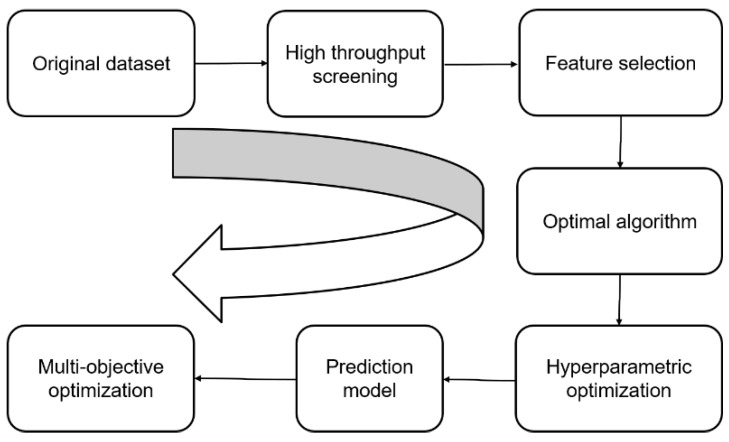
Research route.

To accelerate the search for optimal performance indicators in the search space, this study combined machine learning algorithms with non-dominated genetic algorithms to achieve optimized design of material composition. Figure 2 shows the specific research route. The first step was to collect a large amount of experimental data and construct the original dataset; then, high-throughput screening was conducted on the dataset according to the designed screening strategy to obtain a sub-dataset for learning. Feature selection combined with multiple machine learning regression models was used to compare and find the optimal algorithm, while the hyperparameters in the algorithm were adjusted using Bayesian optimization to establish the final machine learning model. Finally, using the machine learning model’s predictions in the search space as the target, multi-objective optimization was conducted using the NSGA-III algorithm to search for the optimal composition corresponding to the optimal results.

### 2.2. Dataset

**Figure 3:**
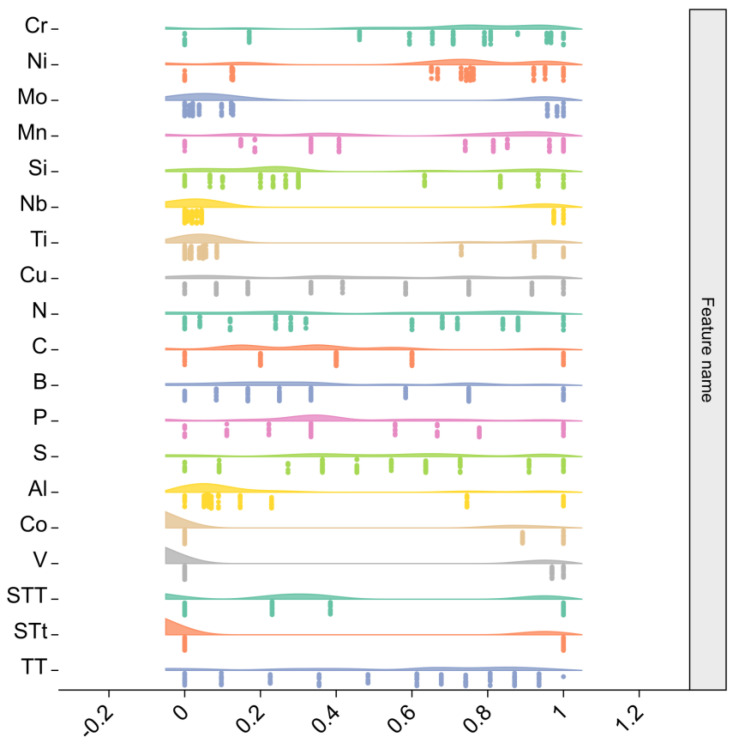
Distribution of original data after feature scaling.

The sample data used in this article were obtained from the Materials Algorithms Project Program Library’s experimental data, which was compiled from the Citrination database [20]. The dataset contains a total of 2085 sample data, including information such as the chemical composition of austenitic stainless steel, solid solution treatment temperature and time, cooling method, melting method, test conditions, mechanical properties, and test sample type. To maintain consistency in all unrelated variables, Table 1 was established to set the screening conditions, ultimately resulting in 132 sample data after screening. Table 2 shows the distribution range of the screened data. To eliminate the influence of differences in the magnitude of different features on machine learning models, the input features were normalized using the feature scaling method. The normalization scaling formula is shown in Equation (1) [21].

(1) $$X^{*}=\frac{X-X_{\min}}{X_{\max}-X_{\min}}$$

where *X*_min_ and *X*_max_ are the minimum and maximum values of the input features. X is the original feature value, and X* is the scaled feature value. Figure 3 shows the distribution of the original data after feature scaling. After feature scaling, all feature variables are distributed between 0 and 1. However, some of the feature data are unevenly distributed with a significant degree of skewness, indicating the original data have discrete values which may have some impact on subsequent analysis.

### 2.3. Model Evaluation and Hyperparametric Optimization

In this study, nine common machine learning algorithms are employed, including decision tree (dtr), random forest (rfr), Adaboost (abg), gradient boosting machine (gbr), extremely randomized trees (etr), bagging regression (br), ridge regression (rdg), least squares orthogonal regression (lso), and XGBoost (xgb). Predictive models are established for the four types of property outputs using compositional features, process features, and testing features as inputs. To compare the performance of different algorithms in the model, fivefold cross-validation is utilized based on the mean absolute error (MAE) and the coefficient of determination (R-squared). Equations (2) and (3) show the calculation formulas for *MAE* and *R*^2^, respectively. The best machine learning algorithm is determined based on the evaluation metrics [22,23].

(2) $$MAE=\frac{1}{n}\sum_{i=0}^{n}\left|y_{i}-f_{i}\right|$$

(3) $$R^{2}=1-{\left(\frac{\sum_{i=1}^{n}\left(y_{i}-f_{i}\right)}{\sum_{i=1}^{n}\left(f_{i}-y_{i,ave}\right)}\right)}^{2}$$

where *n* is the sample size, *y_i_* is the true value, *y_i,ave_* is the mean of the true value, and *f_i_* is the predicted value.

In machine learning, the performance of a model is not only related to the algorithm used but also to the selection of hyperparameters within the algorithm. For instance, the gbr algorithm has many hyperparameters, such as n_estimators, learning_rate, max_depth, subsample, min_samples_split, and min_samples_leaf, whose significances are listed in Table 3. The combinations of these hyperparameters can affect the model’s underfitting or overfitting, so it is necessary to optimize the hyperparameters and determine the best combination. There are generally three methods for hyperparameter optimization in machine learning, namely Grid Search [24], Random Search [25], and Bayesian Optimization [26]. When there are many hyperparameters and a large search space, Grid Search and Random Search may take too long to run, so Bayesian Optimization is often used to optimize hyperparameters. The basic idea of Bayesian optimization [27,28,29] is to determine the optimal hyperparameter combination by continuously evaluating the results of the objective function under each hyperparameter combination. It is a process of model optimization that treats the objective function as a black-box function of the hyperparameters. In each round of iteration, the prior distribution of the existing data will be adjusted to maximize the expected value of the objective function. In-depth analysis is as follows: Let us assume the goal is to find a hyperparameter *θ* ∈ Θ, where Θ represents the space of all possible combinations of hyperparameters. The objective is to discover the optimal hyperparameter θ* that maximizes the score of the objective function *F*(*θ*). It is crucial to first establish an empirical probability model *P*(*F*,*θ*), which represents the joint distribution between the unknown objective constant and the unknown parameter *θ*. Based on Bayesian theory, the posterior probability can be expressed using the Equation (4):

(4) $$P\left(\theta |F\right)=\frac{P\left(F|\theta \right)P\left(\theta \right)}{P\left(F\right)}$$

where *P*(*F|θ*) is the conditional probability density function of the objective function *F* under the hyperparameters *θ,* which describes the probability of finding the given function output *F* after determining the hyperparameters *θ. P*(*θ*) is the “subjective belief” that hyperparameter *θ* possesses without observed data, i.e., the prior distribution, while *P*(*F*) is the normalization constant. After calculating the initial probability of the surrogate model, it is used to guide the specific tuning process, i.e., selecting a new hyperparameter at each iteration and then running the objective function to collect results. By analyzing the information increment obtained from each sampling point, Bayesian optimization can update the transfer probability of the objective function, making it more likely to find the global optimal solution. Finally, after several iterations, the maximum value searched in the observation space is the estimated optimal solution of the objective function *F.*

## 3. Results and Discussion

### 3.1. Model Establishment

**Figure 4:**
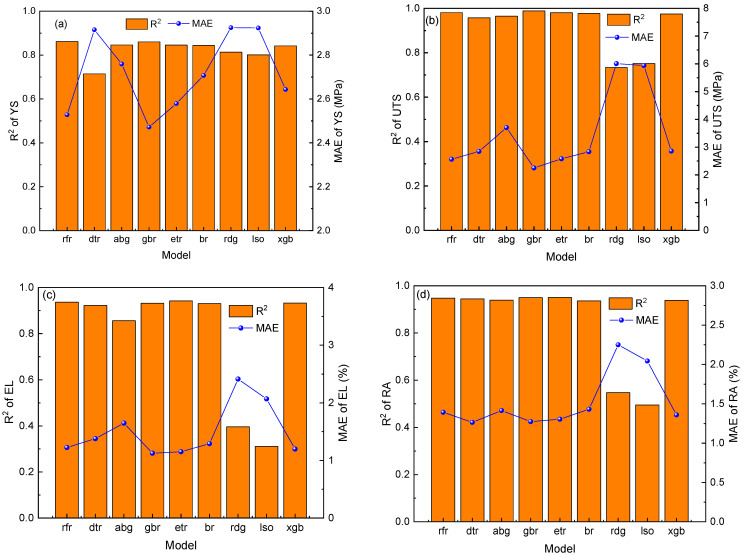
Performance of the dataset on different machine learning models (**a**) YS; (**b**) UTS; (**c**) EL; (**d**) RA.

Figure 4 shows the performance of the dataset on different machine learning models. It can be observed that the gbr algorithm has the highest *R*^2^ value and the lowest *MAE* value, indicating that the gbr algorithm has the best performance in predicting four properties. Therefore, this article will use the gbr algorithm to construct a performance prediction model for further research.

**Figure 5:**
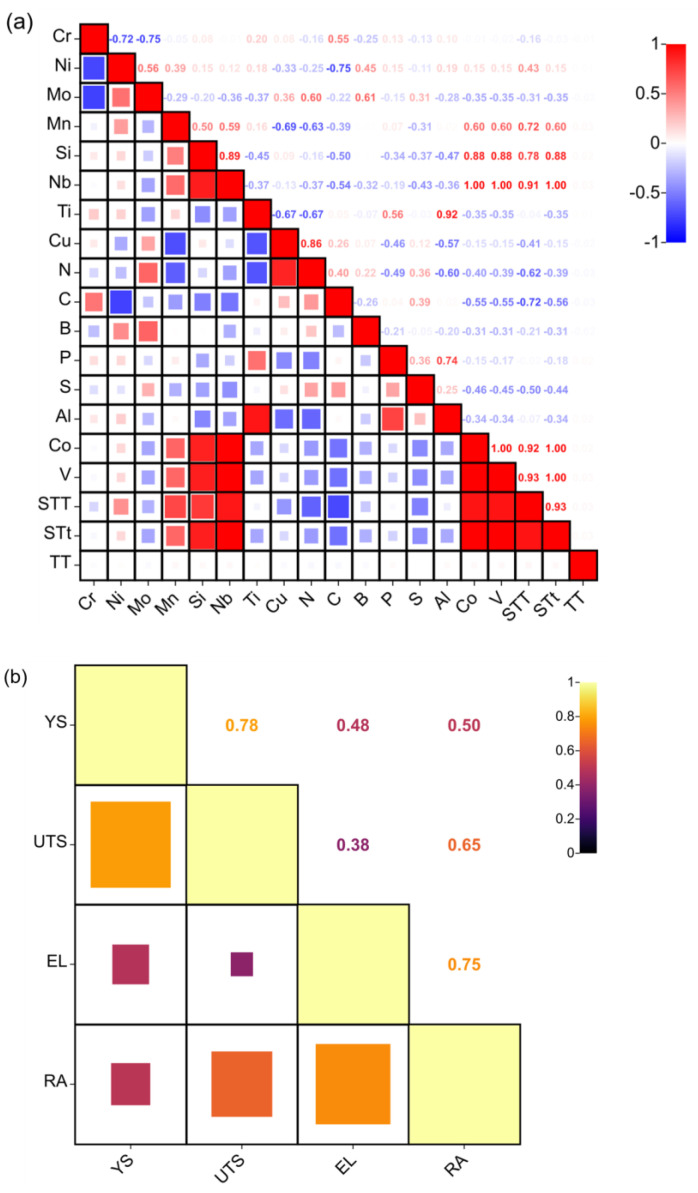
Correlation analysis between data: (**a**) between features; (**b**) between property.

The accuracy of the subsequent machine learning model is directly affected by the number of input features. Therefore, it is necessary to screen the input features and eliminate some redundant features. This study uses correlation filtering to select key feature variables, with the Pearson correlation coefficient [30,31] used to analyze the correlation between the data. When the absolute value of the correlation coefficient between each feature is greater than 0.95, it is generally believed that there is a strong linear correlation between them, and one of the features should be deleted. To determine which feature to delete, a machine learning model is established after each feature is deleted, and the prediction errors of different machine learning models are compared, with the feature exhibiting a large prediction error being deleted. Figure 5a shows the correlation coefficients between the features, with some correlation coefficients between the features being greater than 0.95, requiring screening. Finally, the Co, V, and solution treatment time features are removed. Figure 5b shows the correlation between the performance characteristics. Since there is a test temperature in the input features, it is different from the conventional strength and plasticity, which show a negative correlation. The strength and plasticity in this article exhibit a certain degree of positive correlation.

### 3.2. Hyperparametric Optimization

**Figure 6:**
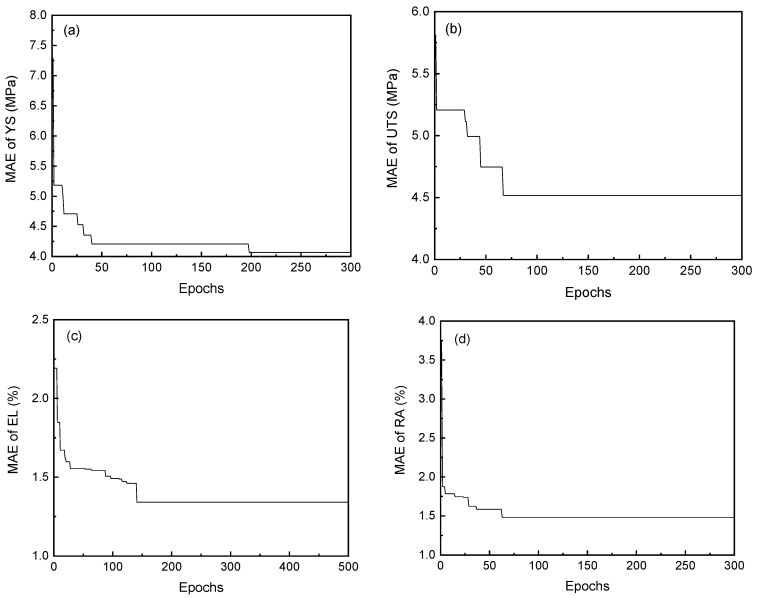
MAE with Bayesian optimization iterations: (**a**) YS; (**b**) UTS; (**c**) EL; (**d**) RA.

**Figure 7:**
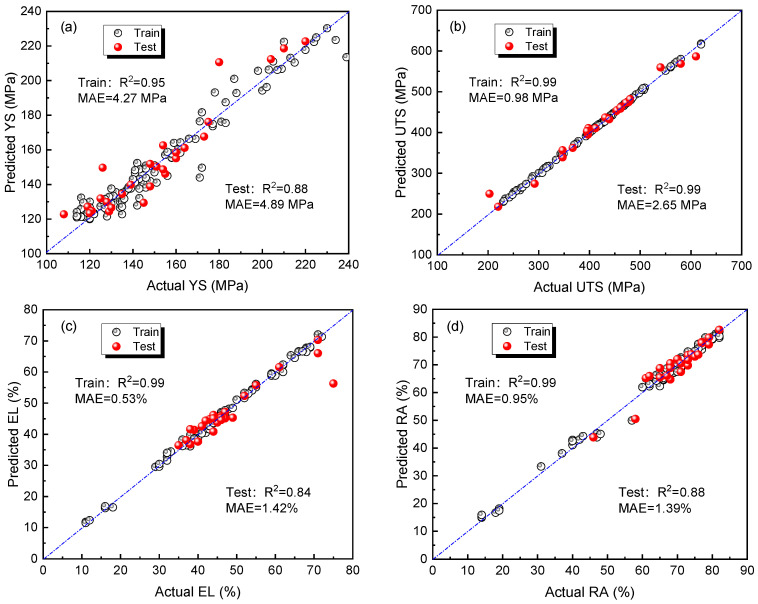
Comparison of predicted and actual values of the model in the training dataset and test set: (**a**) YS; (**b**) UTS; (**c**) EL; (**d**) RA.

After determining the optimal algorithm to be GBR, it is necessary to optimize hyperparameters in GBR to improve model performance. First, before tuning, the original dataset is divided using the hold-out method, with 80% as the training dataset and 20% as the testing dataset. The parameters listed in Section 2.3 are used as tuning targets. The evaluation metric is *MAE*, and fivefold cross-validation is used to fit the training dataset to obtain the best parameter combination for different performance prediction models. Figure 6 shows the change in *MAE* for the four performances with the iteration of Bayesian optimization. With an increase in the number of iterations, the *MAE* of the four mechanical properties gradually becomes stable, indicating that the optimization finally converges. The corresponding optimized hyperparameters are shown in Table 4. Figure 7 shows the comparison between the actual value and the predicted value of the machine learning model established by using the optimized hyperparameters in the training set and the test set. The actual and predicted values of four mechanical properties are mainly distributed along the diagonal line, indicating that the model has good prediction effectiveness. The *R*^2^ of the four mechanical properties in the training set is greater than 0.95, and the *R*^2^ in the testing set is greater than 0.8, which means that the model has good prediction ability for unknown data, providing a foundation for subsequent component optimization.

### 3.3. Interpretable Analysis

**Figure 8:**
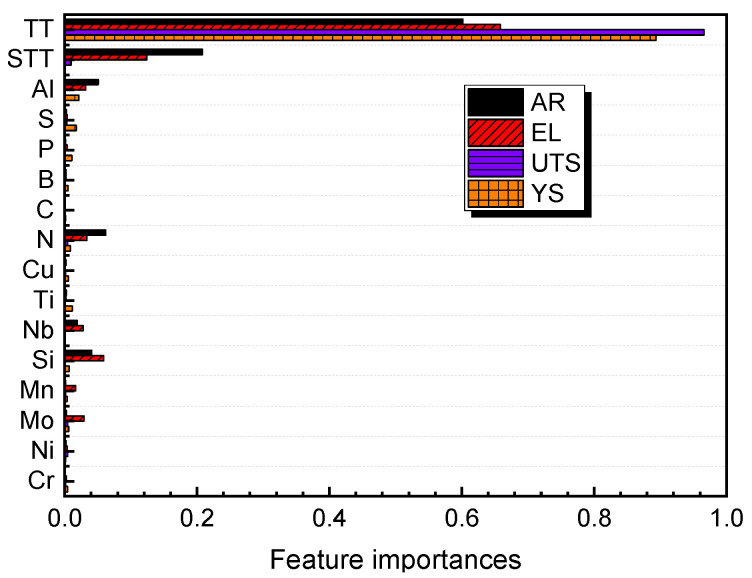
Feature importance ranking of four properties.

**Figure 9:**
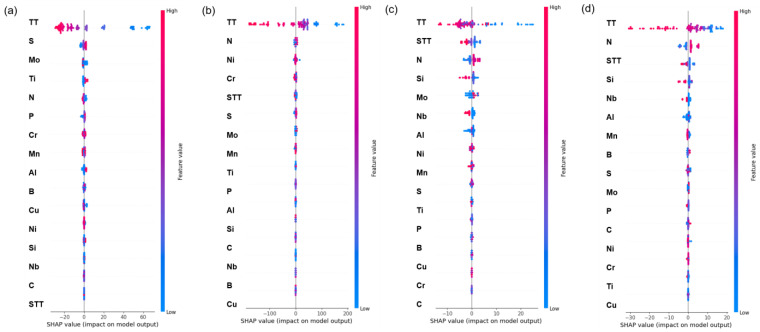
Distribution of features SHAP values: (**a**) YS; (**b**) UTS; (**c**) EL; (**d**) RA.

The above discussion indicates that machine learning models can effectively solve the problem of predicting features and mechanical property. However, since a black box model is established when predicting property, there is a lack of interpretability of how features affect performance. Therefore, it is necessary to analyze the interpretability between features and property. Figure 8 shows the feature importance ranking of the four property features after the machine learning model is established. For the four property features, the test temperature is the most influential among many input features, which is consistent with the conventional view in materials science [32,33,34,35]. However, Figure 8 cannot show how a feature affects performance. Taking the test temperature as an example, it is impossible to know whether the test temperature leads to a decrease or an increase in the final performance. Therefore, the SHAP (Shapley Additive Explanation) value is introduced to analyze how features specifically affect performance [36,37,38,39]. The principle of the SHAP value is based on the concept of “Shapley values” in the theory of positive cooperative games. The mathematical principle of the Shapley value is as follows: for a set of participants with N members, each participant forms a joint combination with the other N−1 participants. The Shapley value assigns the contribution of each participant to different combinations and averages the contribution of all combinations. Specifically, for participant i, the number of times in each combination is divided into two cases: with i and without i. For the contribution of each participant in each combination, the Shapley value calculates the difference between the benefits of the two cases and takes the average to obtain the Shapley value of i. In machine learning models, each feature can be viewed as a participant in a game, and the model prediction result can be viewed as the benefit of the game. The SHAP value is a method for explaining the interpretability of each feature in the actual model prediction result. The calculation of the SHAP value can be based on the model prediction result, input features, and the combination of weighted random sampling points. The contribution of each feature is assigned to each combination, and the SHAP value is finally weighted averaged. Figure 9 shows the SHAP value distribution of each feature for each sample data. Taking Figure 9a as an example, the horizontal axis corresponds to the SHAP value of the sample, and the vertical axis corresponds to each point in the feature. Each point represents a sample. The redder the color of the sample, the larger the corresponding feature value, and the bluer the color, the smaller the corresponding feature value. When the test temperature is small, the SHAP value distribution of the sample is on the positive half-axis, indicating that the test temperature has a positive effect on the yield strength. When the test temperature is large, the SHAP value distribution is on the negative half-axis, indicating that the test temperature has a negative effect on the yield strength. By introducing the SHAP value, the contribution of each feature to the final model prediction value can be effectively analyzed, improving the interpretability of the machine learning model. Figure 9 also shows the effect of alloying elements on the mechanical properties of austenitic stainless steel. For example, Mn element, as a common deoxidizer in iron and steel smelting process, has always existed in stainless steel. At the same time, as an austenite forming element, Mn can stabilize austenite phase and replace part of Ni, but excessive Mn will form MnS inclusions with S, which will reduce the properties of stainless steel [40].

### 3.4. Genetic Algorithm Optimization

To achieve the simultaneous optimization of the four mechanical properties, this study uses the NSGA-III algorithm to screen the composition space of the target component, accelerating the research and development design of high-performance stainless steel. The NSGA-III algorithm [41,42,43,44,45] is an improved version of the NSGA-II algorithm. Compared with NSGA-II, the main improvement of NSGA-III is that it can handle optimization problems with more than three objectives. The core idea of the NSGA-III algorithm is to maintain the diversity of the population by retaining more non-dominated solutions. Specifically, the algorithm divides the population into multiple tiers, with each tier containing a set of dominated solutions, and all solutions in each tier are mutually non-dominated. With this method, NSGA-III can effectively control the diversity of the population and provide better local optimal solutions while ensuring global optimal solutions.

**Figure 10:**
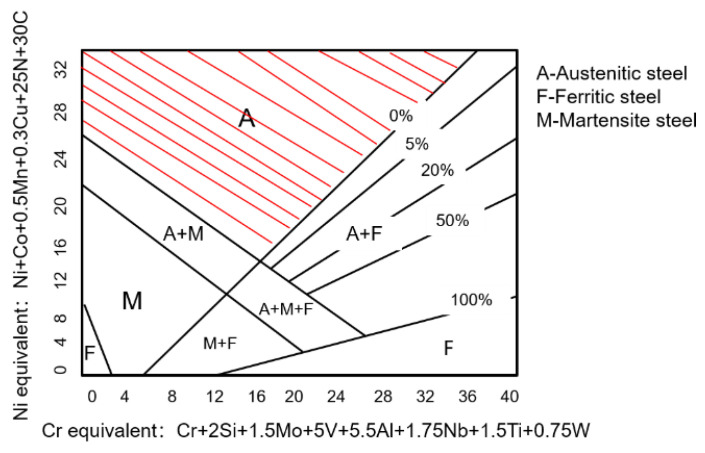
The distribution advantage diagram of the influence of Cr equivalent and Ni equivalent on the microstructure of stainless steel.

After determining the algorithm, the next step is to define the search space and constraints. The main purpose of this study is to optimize the composition of austenitic stainless steel. Therefore, only the composition features change in the search space, and the solution treatment temperature is set at 1343 K, while the test temperature is set at 298 K. The composition features include the alloy content of 14 kinds of steels. Among them, Cr and Ni, which are the highest alloy additions in stainless steel, are limited to 16.42–17 wt.% and 9.8–10 wt.%, respectively, to reduce production costs. The ranges of other alloy contents are the same as the dataset, and the variation step of each alloy component is 0.001. In addition, to ensure that the optimized stainless steel has an austenitic structure, it is necessary to combine the knowledge of experts in the field of materials and constrain the alloy composition. The ferrite forming element (Cr equivalent) represented by Cr and the austenite forming element (Ni equivalent) represented by Ni jointly affect the microstructure of stainless steel, and Equations (5) and (6) show the calculation formulas for Cr equivalent and Ni equivalent, respectively [46]. Figure 10 shows the effect of Cr equivalent and Ni equivalent on microstructure in stainless steel. To constrain the structure of stainless steel to be austenite, the red shadowed area in Figure 10 should be constrained for the Cr equivalent and Ni equivalent.

(5) $${\mathrm{Cr}}_{\mathrm{equivalent}}=\mathrm{Cr}+2\mathrm{Si}+1.5\mathrm{Mo}+5V+5.5\mathrm{Al}+1.75\mathrm{Nb}+1.5\mathrm{Ti}+0.75W$$

(6) $${\mathrm{Ni}}_{\mathrm{equivalent}}=\mathrm{Ni}+\mathrm{Co}+0.5\mathrm{Mn}+0.3\mathrm{Cu}+25N+30C$$

where element symbol (Cr, Si, Mo, …) is the mass fraction of each element.

**Figure 11:**
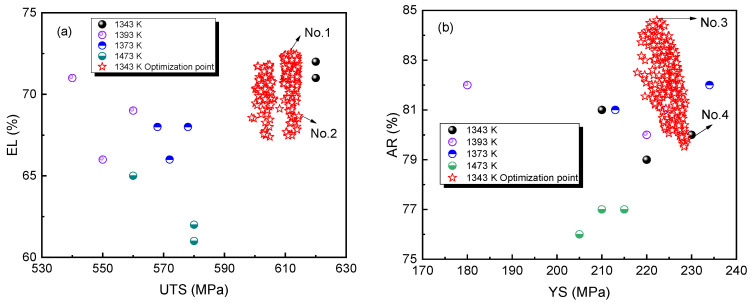
Original and optimized property distributions at test temperature 298 K: (**a**) EL and YTS; (**b**) AR and YS.

After defining the search space and constraints, the parameters of the NSGA-III algorithm are determined. The initial population size for genetic evolution is set to 300, and the number of generations is set to 500. The Polymutation mutation operator is used with a mutation rate of 0.02, and the XOVR crossover operator is used with a crossover rate of 0.9. The chromosome is encoded using the real number encoding RI. Based on the above constraints and composition search range, a selection strategy based on non-dominated sorting and crowding distance is used. After 500 generations of evolutionary search, the non-dominated solution set for four mechanical properties is obtained. Figure 11 shows the corresponding original data distribution and optimized data distribution. Even under the constraint of Cr and Ni content, there are still composition values close to the highest performance, indicating that the composition optimization is effective. Therefore, under the premise of a reliable prediction model, the search strategy of the genetic algorithm is beneficial to the integrated optimization design of multi-objective performance. Table 5 gives the alloy composition corresponding to the maximum value of four mechanical properties in the non-dominated solution set, which is different from any one in the initial dataset. Compared with the typical 18Cr8Ni stainless steel [47] (YS > 205 MPa, UTS > 520 MPa, EL > 40%, AR > 60%), the mechanical properties of the optimized stainless steel are better, but the corrosion resistance may be reduced due to the small addition of alloying elements.

However, further experimental verification is still needed. The main purpose of this study is to explore the feasibility of a multi-objective optimization scheme for austenitic stainless-steel performance and provide optimized composition to guide the next step of experimental design [48,49].

## 4. Conclusions

(1) Nine machine learning algorithms were used to establish prediction models for mechanical properties of austenitic stainless steel. The results show that the gradient boosting regression (gbr) algorithm has the highest prediction accuracy and the best fitting degree.
(2) Bayesian optimization was used to optimize the hyperparameters of the gbr algorithm, and the best parameter combination corresponding to four mechanical properties was obtained. The mechanical properties prediction model established had good prediction accuracy on the test set (YS: R^2^ = 0.88, MAE = 4.89 MPa; UTS: R^2^ = 0.99, MAE = 2.65 MPa; EL: R^2^ = 0.84, MAE = 1.42%; AR: R^2^ = 0.88, MAE = 1.39%).
(3) The feature importance and SHAP value were used to perform interpretable analysis on the performance prediction model. The results indicate that the test temperature is the most important feature affecting the performance, and the high- and low-test temperatures have different positive and negative effects on the performance.
(4) The NSGA-III algorithm was used to optimize the four mechanical properties of austenitic stainless steel, and the constraints and search space were established based on expert knowledge. A new type of austenitic stainless steel with excellent performance was successfully obtained.
(5) The combination of machine learning and genetic algorithm to find the optimal value of performance in the search space can accelerate the research and development efficiency of materials and provide some guidance for the design of new materials.

## Figures and Tables

**Table 1:** Screening conditions.

Features	Variable
Heat treatment	Water cooling after solid solution treatment
Composition	Mass fraction of each element
Steel type	Steel tube
Mechanical properties	Yield strength (YS), ultimate tensile strength (UTS), elongation (EL), and reduction of area (RA)
Test condition	Test temperature
Grain	Grain size
Melting mode	Arc furnace

**Table 2:** Spatial distribution range of filtered dataset.

	Feature Name	Minimum	Maximum	Mean
Composition	Cr content (wt%)	16.42	18.24	17.6113
	Ni content (wt%)	9.8	13.5	12.08947
	Mo content (wt%)	0.02	2.38	0.688626
	Mn content (wt%)	1.47	1.74	1.621221
	Si content (wt%)	0.52	0.82	0.638168
	Nb content (wt%)	0.005	0.79	0.198321
	Ti content (wt%)	0.011	0.53	0.142389
	Cu content (wt%)	0.05	0.17	0.103817
	N content (wt%)	0.013	0.038	0.024901
	C content (wt%)	0.04	0.09	0.059466
	B content (wt%)	0.0001	0.0013	0.059466
	P content (wt%)	0.019	0.028	0.022802
	S content (wt%)	0.006	0.017	0.011573
	Al content (wt%)	0.004	0.161	0.039153
	Co content (wt%)	0	0.37	0.08145
	V content (wt%)	0	0.33	0.007656
Process	Solution treatment temperature/STT (K)	1343	1473	1394
	Solution treatment time/STt (s)	600	1200	742
Test	Test temperature/TT (K)	298	1073	714
Property	YS (MPa)	108	239	153
	UTS (MPa)	203	620	416
	EL (%)	11	75	46
	RA (%)	14	82	66

**Table 3:** Significance of hyperparameters and their impact on the model.

Hyperparameter	Significance
n_estimators	The number of weak learners, that is, the number of subtrees. More trees can improve the model accuracy, but at the same time, it will reduce the running speed of the model, and too many trees may lead to overfitting.
learning_rate	The step size used in each iteration. If the step size is set too large, it may cause the gradient to descend too quickly and fail to converge; conversely, if the step size is set too small, it may take a very long time to reach the optimal result.
max_depth	This parameter limits the depth of the decision tree, controlling the complexity and prediction accuracy of the model. Increasing max_depth will make the model more complex and more prone to overfitting, while smaller values may lead to underfitting.
subsample	The proportion of randomly sampled data for each tree. It is used to control the number of samples in each tree of the training dataset and can be used to solve overfitting problems.
min_samples_split	The minimum number of observations required for a split at an internal node. This parameter can limit the depth of subtree split and prevent overfitting.
min_samples_leaf	The minimum number of samples required to be in a leaf node. Smaller leaf sizes correspond to higher variance and may lead to overfitting problems.

**Table 4:** The best hyperparameters found by Bayesian optimization.

	n_Estimators	Learning_Rate	Max_Depth	Subsample	Min_Samples_Split	Min_Samples_Leaf
YS	223	0.03773	2	0.5	24	1
UTS	347	0.08097	20	1.0	27	5
EL	500	0.03750	8	0.7287	20	4
RA	383	0.09559	2	1.0	20	1

**Table 5:** The alloy composition corresponding to the maximum of four properties (UTS/Mpa EL/% YS/MPa AR/%).

No	Cr	Ni	Mo	Mn	Si	Nb	Ti	Cu	N	C	B	P	S	Al	UTS	EL	YS	AR
1	16.98	9.80	0.121	1.481	0.597	0.375	0.021	0.051	0.026	0.090	0.00039	0.023	0.00604	0.0138	611	72.5	221	81.5
2	16.99	9.98	0.0395	1.568	0.598	0.276	0.521	0.052	0.025	0.043	0.00113	0.022	0.01700	0.1438	614	68.7	223	81.9
3	16.99	9.96	1.020	1.471	0.597	0.116	0.103	0.050	0.013	0.041	0.00092	0.022	0.01263	0.0141	601	70.5	224	84.6
4	16.84	9.92	0.181	1.671	0.597	0.295	0.529	0.050	0.026	0.042	0.00017	0.023	0.00625	0.0142	603	68.2	229	79.8

## Data Availability

The data presented in this study are available upon request from the corresponding author.
